# Coenzyme Q_10_ and Cardiovascular Diseases

**DOI:** 10.3390/antiox10060906

**Published:** 2021-06-03

**Authors:** Francisco M. Gutierrez-Mariscal, Silvia de la Cruz-Ares, Jose D. Torres-Peña, Juan F. Alcalá-Diaz, Elena M. Yubero-Serrano, José López-Miranda

**Affiliations:** 1 Lipids and Atherosclerosis Unit, Unidad de Gestión Clínica de Medicina Interna, Maimonides Institute for Biomedical Research in Córdoba (IMIBIC), Reina Sofia University Hospital, University of Córdoba, 14004 Córdoba, Spain; francisco.gutierrez@imibic.org (F.M.G.-M.); scruz@uco.es (S.d.l.C.-A.); h42topej@uco.es (J.D.T.-P.); juanf.alcala.sspa@juntadeandalucia.es (J.F.A.-D.); elena.yubero@imibic.org (E.M.Y.-S.); 2 CIBER Fisiopatología Obesidad y Nutrición (CIBEROBN), Instituto de Salud Carlos III, 14004 Córdoba, Spain

**Keywords:** coenzyme Q_10_, ubiquinone, ubiquinol, cardiovascular diseases

## Abstract

Coenzyme Q_10_ (CoQ_10_), which plays a key role in the electron transport chain by providing an adequate, efficient supply of energy, has another relevant function as an antioxidant, acting in mitochondria, other cell compartments, and plasma lipoproteins. CoQ_10_ deficiency is present in chronic and age-related diseases. In particular, in cardiovascular diseases (CVDs), there is a reduced bioavailability of CoQ_10_ since statins, one of the most common lipid-lowering drugs, inhibit the common pathway shared by CoQ_10_ endogenous biosynthesis and cholesterol biosynthesis. Different clinical trials have analyzed the effect of CoQ_10_ supplementation as a treatment to ameliorate these deficiencies in the context of CVDs. In this review, we focus on recent advances in CoQ_10_ supplementation and the clinical implications in the reduction of cardiovascular risk factors (such as lipid and lipoprotein levels, blood pressure, or endothelial function) as well as in a therapeutic approach for the reduction of the clinical complications of CVD.

## 1. Introduction, CoQ_10_ in Cardiovascular Diseases

Coenzyme Q_10_ (CoQ_10_) was isolated by Festenstein et al. (1955) and Crane et al. (1957). Chemically, it is a lipid-soluble, biologically active quinone with a benzoquinone ring and an isoprenoid sidechain containing 10 residues of isoprenoid. The main function of CoQ_10_ is to take part in the mitochondrial electron transport chain, where it carries electrons from complex I and II to complex III [1,2]. During the decades since it was first described, multiple functions have been attributed to CoQ_10_ such as to control the cellular redox state both by its antioxidant properties and by the generation of oxidant signals as well as a role in proton gradient formation at the endomembrane and plasma membrane, which contributes to control the membrane structure and phospholipid status [3,4,5].

**Figure 1:**
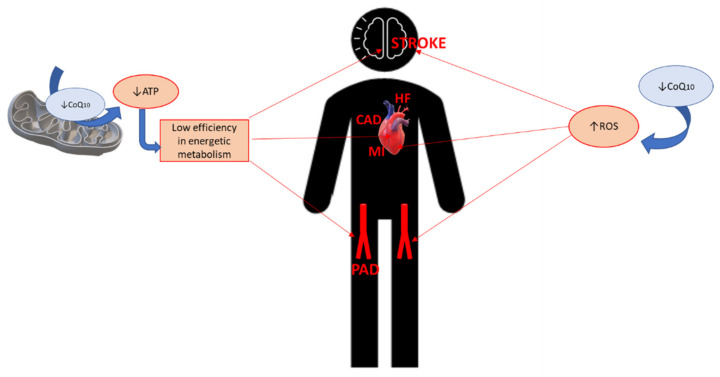
Pathogenic background of coenzyme Q10 in cardiovascular diseases. CoQ_10_: Coenzyme Q_10_; ATP: Adenosine triphosphate; CAD: Coronary artery disease; HF: Heart failure; MI: Myocardial infarction; PAD: Peripheral artery disease; ROS: Reactive oxygen species.

The main function of CoQ_10_ has one important consequence for energy metabolism, since better, more efficient electron transport in the inner membrane at the mitochondria leads to more abundant production of ATP. This is a highly relevant factor, for instance, for the cardiac muscle and the correct functioning of the heart. This direct effect of CoQ_10_ on CVDs such as heart failure (HF) or myocardial infarction is accompanied by the crucial action of CoQ_10_ as a potent antioxidant due to its coexistent redox forms (ubiquinone, semi-ubiquinone and ubiquinol), which act in the mitochondrial membrane, other cell membranes, and in plasma and cytoplasm. These antioxidant properties of CoQ_10_ act not only on the electron transport chain in the mitochondria, but also in recycling other antioxidants such as vitamin C or vitamin E. Along with its influence on the efficiency of energetic metabolism, these other functions of CoQ_10_ have an important impact on cardiovascular health in humans, affecting the endothelial and vascular system, which in turn influences the incidence, etiology, and progression of other CVDs such as coronary artery disease (CAD) (Figure 1). For these reasons, we aimed to review the latest publications on the effect of CoQ_10_ on cardiovascular health. In this review, we explore and analyze our current knowledge of this issue and its future perspectives.

## 2. The Biology of CoQ_10_

CoQ_10_ is ubiquitous in all cell membranes, and is endogenously biosynthesized in all tissues of the organism. A minor proportion of comes from dietary sources. However, from a clinical point of view, it is noteworthy that endogenous production deficiency takes part in the pathophysiology of different diseases, being that its biosynthesis is significantly reduced with aging.

**Figure 2:**
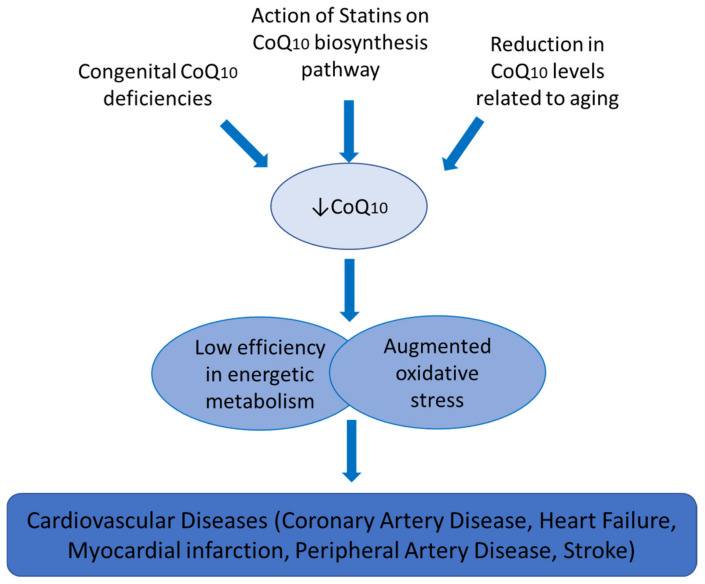
Coenzyme Q_10_ and cardiovascular diseases.

Endogenous CoQ_10_ biosynthesis is carried out by a pathway involving at least 11 genes, named *COQ* genes, which are well-conserved among species [6]. The first step involves the benzoquinone ring being synthesized as 4-hydroxybenzoato and the isoprenoid side chain precursor, acetyl-CoA [7,8]. In the context of CVDs, there is an important connection between CoQ_10_ biosynthesis and the action of one of the most common lipid-lowering drugs, statins. Cholesterol biosynthesis shares a common pathway with CoQ_10_ endogenous biosynthesis, which means that the use of statins in the treatment of hypercholesterolemia leads to a reduction in the synthesis of CoQ_10_. This, in turn, results in a reduction in the disposable supply of this compound, reducing the efficacy of energetic metabolism and its implications in cardiac metabolism and redox status (Figure 2). Regarding the distribution of CoQ_10_ in the organism, it is known to be present in varying amounts in different organs. Its levels range from 8 μg/g in the lung to 114 μg/g in the heart. Generally, it is present more in tissues with high-energy requirements or metabolic activity such as the heart, kidney, liver, and muscle [9]. However, CoQ_10_ levels are disrupted by health and disease status. It has been reported that lesser amounts of CoQ_10_ are observed in Alzheimer’s disease, cardiomyopathies, or type 2 diabetes mellitus compared to healthy people [10,11].

Another important factor in the biology and metabolism of CoQ_10_ is the bioavailability and distribution of the different formulations of this compound when administered orally. This is determined by its lipophilic characteristics, which make it extremely insoluble in water. For this reason, the typical regimen of oral administration of CoQ_10_ takes advantage of when lipid-rich foods are consumed [12]. In this context, many of the studies published have failed to represent the real amount of CoQ_10_, which reaches the tissues, showing, in most cases, the levels of this quinone measured in plasma, as a surrogate measurement of the CoQ_10_ already ingested.

Despite these recommendations, research on CoQ_10_ absorption and bioavailability varies, and is dependent on the type of CoQ_10_ preparation used [13,14]. Many formulations have been developed to improve CoQ_10_ solubility in the organism. Recent new formulations for CoQ_10_ are based on enhancing its water-solubility, as in the cases of Qter or Ubisol-Q_10_. Ubisol-Q_10_ is a nanomiscelle formulation that appears to be water-soluble containing CoQ_10_, where solubilization is achieved due to the amphipathic properties of polyetilenglycol-derivatized α-tocopherol, which allows for the formation of stable and water-soluble nanomicelles [15]. Q-ter is a supplement consisting of copovidone, which acts as a carrier, CoQ_10_, and glycine, which works as a catalyst. This composition makes Q-ter 200-times more soluble in water than pure CoQ_10_ [16].

Other efforts have been focused on discovering analogues of CoQ_10_ with greater solubility and antioxidant effects such as MitoQ. MitoQ is also known as MitoQuinone (Phosphonium [10-(4,5-dimethoxy-2-methyl-3,6-dioxo-1,4-cyclohexadien-1-yl)decyl] triphenyl-,mesylate) and is a mitochondria-targeted antioxidant composed of a quinone moiety attached to triphenylphosphonium (TPP) via a 10-carbon alkyl chain. The lipophilic nature of TPP allows the antioxidant to be allocated in the mitochondrial matrix and accumulate there [17]. However, there is very little literature on the use of these new formulations of CoQ_10_ in randomized controlled trials (RCT) in CVDs, and most of them are in the field of neurodegenerative diseases.

## 3. Methodology of Review

CoQ_10_ has been extensively reviewed over the years. In this review, we planned to focus on CVDs and the most relevant findings published in the past five years. We performed a search on PubMed on 31 January 2021 with the keywords “Coenzyme Q_10_ and cardiovascular diseases”. From the results of the search, we selected those diseases which were more commonly represented among the studies from 2015, had been performed in humans, and were predominantly in RCT. After revisiting this bibliography, we decided to include the recent publications of studies on the influence of CoQ_10_ on cardiovascular risk factors (dyslipidemia, endothelial dysfunction, and hypertension), HF, myocardial infarction, stroke, peripheral artery disease (PAD), and CAD. For each of these items, we performed a new search on PubMed with “CoQ_10_/coenzyme Q_10_/ubiquinone cardiovascular risk factors/dyslipidemia/endothelial dysfunction/hypertension/heart failure/myocardial infarction/stroke/peripheral artery disease/coronary artery disease”. From the publications obtained in these searches, we selected those with the highest relevance.

## 4. CoQ_10_ and Cardiovascular Risk Factors

Different studies have examined the efficacy of CoQ_10_ supplementation in the prevention of CVD through the reduction of cardiovascular risk factors (such as lipid and lipoprotein levels, blood pressure or endothelial function) with the aim of improving patient health and quality of life.

### 4.1. Dyslipidemias

Although the effect of CoQ_10_ supplementation on lipid and lipoprotein levels is quantitatively small, different clinical studies, meta-analysis, and systematic reviews have supported the beneficial effects in several types of patients for different CVD risks. In a meta-analysis conducted by Sharifi et al. [18], the authors concluded that administration of CoQ_10_ significantly reduced triglyceride (TG) concentrations, but not total cholesterol and LDL-cholesterol levels in patients with metabolic disease. Similarly, Jorat et al. [19] found in a meta-analysis that CoQ_10_ supplementation decreased total cholesterol and increased HDL-cholesterol levels in patients with CAD. However, in a randomized, double-blind, placebo-controlled study performed in obese participants, CoQ_10_ supplementation (200 mg/d for 12-weeks) did not significantly affect lipid profiles [20]. In dyslipidemic subjects, the administration of 120 mg/d of CoQ_10_ for 24-weeks reduced TG and LDL-cholesterol concentrations and increased apolipoprotein A-1 compared to the placebo [21]. Results of a double-blinded randomized clinical trial using 200 mg/d of CoQ_10_ for 12-weeks showed a significant increase in HDL-cholesterol and a significant decrease in total cholesterol/HDL-cholesterol ratio in patients with hyperlipidemia and myocardial infarction [22]. The total cholesterol/HDL-cholesterol ratio is described as a significant predictor of cardiovascular events and a therapeutic target in high-risk patients [23]. In patients with type 2 diabetes and hyperlipidemia, CoQ_10_ increased the total cholesterol and LDL-cholesterol levels, but had no effect on HDL-cholesterol compared with the placebo [24]. 

In the context of CAD, a recent systematic review found that CoQ_10_ supplementation significantly decreased total cholesterol and increased HDL-cholesterol levels without affecting TG and LDL-cholesterol [19]. Considering the fact that the use of statins to decrease cholesterol synthesis also affects CoQ_10_ levels, the use of exogenous CoQ_10_ supplementation has been considered to preserve plasma CoQ_10_ levels in patients treated with these therapeutic compounds (such as patients with CAD and HF) [25]. The combination of CoQ_10_ with statins has been recently proposed to benefit hypercholesterolemic patients with chronic heart failure [26,27]. In this context, in these patients, the co-administration of CoQ_10_ and statin therapy is found to be highly recommendable to avoid myopathic side effects as well as enhancing antioxidant enzymes activities and reducing inflammation [28,29]. An overview of the main evidence related to CoQ_10_ supplementation studies, referred to in the text, is shown in Table 1 [16,17,18,19,20,21,22,23,24,25,26,27,28,29,30,31,32,33,34,35,36,37,38,39,40,41,42,43,44,45,46,47,48,49,50,51,52,53,54,55,56,57,58,59,60,61,62,63,64,65,66,67,68,69,70,71,72,73,74,75,76,77].

It has been suggested that CoQ_10_ could act, in the improvement of lipid and lipoprotein profiles, through the activation of the gene expression of peroxisome proliferator-activated receptor-gamma (PPAR-γ) [30], a nuclear receptor protein that regulates pathways related to insulin, lipid metabolism, and inflammation [31] or via the AMP-activated protein kinase/protein kinase C/NADPH oxidase signaling pathway, which suppresses oxidized LDL-induced endothelial oxidative injuries by modulating oxidized low-density lipoprotein receptor 1-mediated reactive oxygen species (ROS) generation [32].

### 4.2. Hypertension

Hypertension is a key risk factor for almost all CVDs. Although many pharmacological therapies have provided a beneficial effect in lowering blood pressure with a modest decrease in cardiovascular mortality, hypertension remains prevalent [33,34]. Therefore, the effect of CoQ_10_ on blood pressure has been studied in different controlled intervention studies in human subjects, with a range of CoQ_10_ doses from 100 mg to 200 mg/day [35,36,37]. These studies found that patients treated with CoQ_10_ decreased both systolic and diastolic blood pressure, without significant side effects [38]. In a systematic review performed in the context of primary prevention, the authors found that CoQ_10_ supplementation, without lifestyle intervention, produced a significant reduction in systolic blood pressure without an improvement in other CVD risk factors [39]. In a randomized, double-blinded controlled clinical trial conducted in patients with hyperlipidemia and myocardial infarction, CoQ_10_ supplementation (200 mg/d) for 12-weeks led to a decrease in both systolic and diastolic blood pressure [22]. In fact, a recent review, in the context of primary prevention, argued that CoQ_10_ could be used as an effective antihypertensive agent with a capacity of lowering blood pressure to 11/7 [40]. However, in several studies conducted in patients with ischemic left ventricular systolic dysfunction, type 2 diabetes mellitus or obesity, but without hypertension, supplementation of CoQ_10_ did not alter blood pressure [20,41].

Strong evidence also points to a direct effect of CoQ_10_ on the endothelium by improving vascular smooth muscle activity, counteracting vasoconstriction, and lowering blood pressure [42]. Moreover, CoQ_10_ is also thought to provide a protective role in hypertension, acting indirectly through its ability to prevent oxidative stress, nitrative stress, and inflammation, resulting in a recoupling of endothelial nitric oxide synthase (eNOS) [43].

### 4.3. Endothelial Dysfunction

Endothelial dysfunction, a main mechanism underlying the development of arteriosclerotic disease, is considered a significant predictor of cardiovascular risk [44,45]. The effect of CoQ_10_ supplementation on the modulation of endothelial function has been evaluated in patients with type 2 diabetes mellitus, CAD, or in elderly people [46,47,48]. These results showed that flow-mediated dilation, or nitroglycerin-mediated dilation and the extracellular superoxide dismutase activity increased in most of the subjects treated with CoQ_10_, attributing this effect to its antioxidant and anti-inflammatory activity [35,48,49]. Although the mechanisms of CoQ_10_ in modulating endothelial function are still unclear, it has been suggested that it could be partly attributed to its capacity for reducing oxidative stress and inflammation, particularly in myocardial and endothelial cells [50] and decreasing the rate of activation of NO to peroxynitrite by superoxide radicals, which could improve both vascular tone and endothelial function [51]. CoQ_10_ may also affect vascular function indirectly via the inhibition of oxidative damage to LDL [43]. In fact, in patients with HF, treatment with CoQ_10_ (400 mg/d for three months) resulted in significant improvements in peripheral endothelial function, determined by the reactive hyperemia index, accompanied by low levels of oxidized-LDL [52]. CoQ_10_ has also been shown to improve endothelial function in patients with CAD [48] and type 2 diabetes mellitus [53].

Moreover, it has also been suggested that the protection of the endothelial function through capturing blood plasma CoQ_10_ and thus reducing oxidative stress could play a key role in the beneficial effect of plasma CoQ_10_ levels in CVD [46]. In fact, patients with moderate dyslipidemia and endothelial dysfunction showed an improvement in their cardiovascular status after treatment with CoQ_10_ [54]. Furthermore, the combined action of CoQ_10_ and anti-atherogenic drugs such as statins improved endothelial dysfunction [55].

## 5. CoQ_10_ and CVDs

Cardiac tissues of patients with CVD (heart failure, angina pectoris, coronary artery disease or cardiomyopathy) exhibited a strong CoQ_10_ deficiency [56,57]. Nutraceuticals have been shown as effectively capable of reducing the burden of the atherosclerosis process and CVD development, as already demonstrated in the literature [58]. In this context, different studies have analyzed the effect of CoQ_10_ supplementation on CVD as a therapeutic approach to reduce the clinical complications of CVD.

### 5.1. CoQ_10_ in Coronary Artery Disease

CAD is the most common type of heart disease. It is also known as coronary heart disease or ischemic heart disease. In its etiology, CAD is caused by the formation of an atherosclerotic plaque in the walls of the arteries that supply blood to the heart (coronary arteries) and other parts of the body. Due to the known involvement of oxidative damage in the etiology of atherosclerosis, CoQ_10_ has been thought to have potential benefits in the amelioration of CAD. Heng Lu et al. recently showed, in a correlation analysis of metabolic pathways integrated as a unit to co-express susceptibility genes with CAD, that the canonical metabolic pathway of biosynthesis of CoQ_10_, specifically the genes *COQ2* and *COQ5*, were linked to the susceptibility of CAD [59]. The *COQ2* gene encodes 4-hydroxybenzoate polyprenyltransferase enzyme, which condenses decaprenyl pyrophosphate and 4-OH-benzoate into decaprenyl-OH-benzoate [5,60]. The *COQ5* gene encodes the enzyme that catalyzes the only C-methylation step involved in the synthesis of CoQ_10_ [61]. Due to the action of CoQ_10_ in energy metabolism and its relation to coronary revascularization, some authors consider that low levels of CoQ_10_ should be considered as a risk factor for CAD [62,63].

In a systematic review carried out by Jorat et al. in 2019, the authors looked for evidence of effects of CoQ_10_ supplementation on inflammation and oxidative damage in CAD patients [64]. Thirteen out of 912 potential RCT found in the literature were analyzed and it was found that CoQ_10_ supplementation significantly increased the levels of the antioxidant enzymes superoxide dismutase (SOD) and catalase, and significantly reduced malondialdehyde (MDA), a marker of lipid peroxidation. Since CAD patients are considered to have a chronic systemic inflammation status, this recent meta-analysis demonstrated the potential benefits of CoQ_10_ supplementation for ameliorating inflammation and oxidative damage in these patients. However, this analysis failed to demonstrate the effects of CoQ_10_ on C-reactive protein, tumor necrosis factor α, interleukin-6, and glutathione peroxidase levels among patients with CAD, which have been described previously [28]. The dosage range presented in the RCT included in the analysis was from 60 to 300 mg/day of CoQ_10_ with a follow-up of up to 48 weeks. However, no information was reported regarding the formulation used for the supplementation. Since it has a great impact on the bioavailability of CoQ_10_, and there seems to be a consensus that at least 200 mg/day is needed to have an impact on rising plasma CoQ_10_ levels [5], there is a need for a RCT with these characteristics to elucidate the real effects of CoQ_10_ on inflammatory and oxidative damage in these patients.

### 5.2. CoQ_10_ in Heart Failure

Heart failure (HF) is defined as “a complex clinical syndrome that can result from any structural or functional cardiac disorder that impairs the ability of the ventricle to fill or eject blood” [65]. Despite improvements in the prevention and treatment of HF, mortality rates from HF are over 10% per year, and even reach 20% to 50% in some settings [66]. In HF, the heart muscle exhibits reduced adenosine triphosphate synthesis, increased production of ROS, and a deflection of the calcium exchange, mainly due to inefficient electron transport chain activity. Moreover, in patients with HF, the severity of disease is correlated with CoQ_10_ deficiency [67]. In this context, and since CoQ_10_ plays a key role in cell energetics in the mitochondria, it is plausible to consider the potential benefits of CoQ_10_ supplementation as a therapeutic option for HF patients. It has also been demonstrated that CoQ_10_ prevents senescence and dysfunction in vascular endothelial cells caused by oxidative damage [51]. For this reason, supplementation with CoQ_10_ has been suggested to prevent not only HF but also hypertension and endothelial dysfunction (see Section 4.3).

Over the last few years, several clinical studies have investigated the possibility of using CoQ_10_ to prevent HF and improve the symptoms of this disease. In one of the most important clinical studies related to this research area, the Q-SYMBIO study, it was shown that in a study including 420 patients with moderate or severe HF (202 with 300 mg of CoQ_10_ supplementation and 218 with placebo), there was a reduction in the rate of major adverse cardiac events, cardiovascular mortality, all-cause mortality, and incidence of hospital stays for HF, after 2-years, compared to those patients treated with the placebo [68]. However, the Q-SYMBIO study has been criticized, for instance, for not reaching the planned number of patients (*n* = 550) in eight years of recruitment. Indeed, in a sub-group analysis of this study recently published, the researchers observed improvements in major clinical endpoints, with an increase in left ventricular ejection fraction in the European population of the study, which was not found in the larger cohort. The authors declared that this subpopulation of the study showed a higher adherence to the recommended medical and device therapies compared to the whole population. Furthermore, the effects found in this study were confirmed in a subsequent meta-analysis of 14 RCTs including 2149 patients, although no significant differences were observed in the endpoint of left ventricular ejection fraction between the group that received treatment and the group receiving a placebo [69]. However, short-term CoQ_10_ supplementation provided no additional benefits in improving left ventricle diastolic function in 28 patients with HF with preserved ejection fraction [70]. In general, the heterogeneity in the populations, designs, follow-up durations, doses administered, and study outcomes make it difficult to extract a clear effect of CoQ_10_ in HF. Nevertheless, there seems to be a consensus that the beneficial effects attributable to CoQ_10_ in HF are related to its important role as an electron carrier in mitochondria, increasing bioenergetics and preventing oxidative damage in the failing myocardium. However, changes in the antioxidant systems in HF support the idea that CoQ_10_ may improve the outcome and quality of life and may decrease morbidity and mortality.

### 5.3. CoQ_10_ in Myocardial Infarction

Myocardial infarction occurs due to myocardial cell death caused by prolonged ischemia [71]. The pathologic processes underlying this disease are linked to a high oxidative stress that leads to reperfusion-induced free radical damage, lipid peroxidation, and decreased energy production, in which CoQ_10_ deficiency could play a role [72,73]. In fact, recent evidence has shown that maintaining high endogenous levels of CoQ_10_ in plasma, in patients with myocardial infarction, is related to a better recovery of left ventricular function [74]. In this context, another possible use for CoQ_10_ supplementation could be to restore tissue CoQ_10_ deficiency in the myocardium after myocardial infarction. In a recent RCT, the authors showed an improvement of quality of life in patients with myocardial infarction using the MacNew QoL questionnaire, after 3-months of supplementation with 150 mg/d of CoQ_10_ plus 200 mg/d of L-carnitine [75]. Besides a reduction in blood pressure, LDL-cholesterol/HDL-cholesterol and total cholesterol/HDL-cholesterol ratios in patients who presented hyperlipidemia but also myocardial infarction, CoQ_10_ supplementation (200 mg/day for 12-weeks) also decreased inflammatory status (serum ICAM-1 and IL-6 levels) [22,76]. In diabetic patients with CAD, CoQ_10_ supplementation also produced an anti-inflammatory effect despite the fact that no improvement was observed in cardiometabolic markers, suggesting that the presence of type 2 diabetes could infer different underlying pathogenic mechanisms in myocardial infarction [77].

Regarding the possibility of using CoQ_10_ supplementation to prevent cardiac remodeling in patients with myocardial infarction, in another study, 24-weeks of supplementation of CoQ_10_ (120 mg/day) showed the maintenance of the sphericity index with diminished alteration of wall-thickening abnormality at the infarct site, compared to the placebo, in patients affected by persistent left ventricular dysfunction [78]. Moreover, in a recent study performed in patients with myocardial infarction using 120 mg of CoQ_10_/day for 24-weeks, a protective role against left ventricular remodeling was observed in patients with persistent left ventricular dysfunction [79].

### 5.4. CoQ_10_ in Peripheral Artery Disease

PAD is a common CVD characterized by the formation of atherosclerotic plaque in the leg arteries, which causes attenuated blood flow and reduced perfusion in the lower extremities [80]. PAD can severely impact quality of life as functional ability is compromised as the disease progresses. Among the risk factors for the incidence of this disease are smoking, diabetes mellitus and CAD, and age, with approximately 20% of the population over 60 years presenting some degree of the condition. Symptoms often include claudication (leg pain during walking) and foot ulcers, which, if severe, may require revascularization procedures or leg amputation [81].

In PAD, increased ROS appears as one of the mechanisms underlying atherosclerosis and oxidative stress damage in the skeletal muscle [82]. At this point, maintaining an effective function of the mitochondria in the vasculature is a key factor that controls ROS production and NO bioavailability. In this context, mitochondria could be seen as a novel therapeutic target to improve vascular function, oxygen transfer, and utility capacity in the lower extremity, with a potential role in reducing leg pain and improving quality of life in PAD patients [83]. Despite these premises, few studies have been published that aim to evaluate the effect of CoQ_10_ supplementation in the amelioration of PAD. Among recent publications, only one study with 11 PAD patients is worth mentioning. In a randomized, placebo-controlled, crossover study design, patients received a dose of 80 mg of MitoQ, an analogue of CoQ_10_ [83]. The study aimed to understand the roles of the vascular mitochondria in PAD in vivo by examining the impacts of acute MitoQ intake on endothelial function, blood pressure, arterial stiffness, walking capacity, and oxygen utility capacity in patients with PAD. The authors found, for the first time, an improvement in brachial artery endothelial function after the intake of MitoQ, highlighting the key role that vascular mitochondria plays in endothelial function in this kind of patient. Regarding the molecular mechanisms behind these effects, Park S.Y. et al. showed an increase in SOD, the first line of the endogenous antioxidant defense system, which could reduce mitochondrial-derived ROS in the vasculature. In line with these findings, it seems to be of importance to confirm the effects of CoQ_10_ in the amelioration of PAD in larger cohorts. Despite the lack of clinical trials proving the benefits of CoQ_10_ supplementation, a recent review summarized the nutrients that may be at risk of depletion in PAD patients treated with lipid-lowering, antiplatelet, antihypertensive, and antidiabetic drugs. Among them, the authors identified as potentially at risk from CoQ_10_, vitamin C, zinc, and vitamin B12 [84]. As these medications are frequently prescribed to PAD patients, the authors recommend that CoQ_10_, zinc, and vitamin B12 levels should be routinely monitored to prevent and correct nutrient deficiencies associated with the multiple-drug therapy for this condition. This leads to the possible need for dietary interventions, and the possible supplementation with these nutrients in PAD patients.

### 5.5. Stroke

Stroke occurs when the blood supply to part of the brain is interrupted or reduced, preventing the brain tissue from receiving oxygen and nutrients. Brain cells can only survive a few minutes without this supply, and this leads to problems of ischemic brain damage, which is extremely common in modern health care. In addition, ischemic stroke has major socio-economic consequences, leading in some cases to disability in working-age people. Oxidative stress has been described as one of the pathological mechanisms involved in cerebral ischemia [85], where a disbalance among oxidants and antioxidants causes further damage to cell structure and function [86]. Despite the evidence of the CoQ_10_ functions described, to the best of our knowledge, there are no recent publications in the literature regarding clinical studies in humans considering the beneficial effect of CoQ_10_ supplementation to ameliorate the symptoms and consequences of stroke, although there is one clinical study that aimed to associate CoQ_10_ levels with clinical neurological outcomes in acute stroke patients [87]. This study found that stroke patients had significantly lower serum levels of CoQ_10_ and SOD compared to the controls, and higher serum MDA levels, in a cohort of 76 patients and 34 healthy individuals. The authors suggest that a decrease in serum CoQ_10_ level exerts a detrimental effect on neurological outcomes following acute ischemic stroke, as measured by the National Institute of Health Stroke Scale and a modified Rankin Scale. Therefore, it can be suggested that preserving the antioxidant functionality of CoQ_10_ could be a potential strategy in treating this pathology. On the other hand, there is a more extensive bibliography, compared to human studies, on the influence of CoQ_10_ as a treatment in animal models. Although the scope of this review only includes human studies, it is interesting to note that Nikolaevna et al. recently found that intravenous administration of CoQ_10_ led to a decrease in rat mortality rate, improvement in neurological status and decrease in the brain necrosis area in acute and delayed periods after cerebral ischemia [88]. Due to the lack of more clinical trials on the effect of pre- or post-treatment with CoQ_10_ in strokes in larger human studies, we can only highlight the promising impact this type of treatment could have on stroke patients.

## 6. Conclusions

Although the importance of CoQ_10_ can mostly be attributed to its function as an essential molecule for energy transduction in mitochondria, new findings support its relevant function as an antioxidant, not only in mitochondria, but also in other cell compartments and tissues in the organism as well as in plasma lipoproteins. Endogenous CoQ_10_ biosynthesis supplies sufficient levels of this quinone in disease-free individuals. However, CoQ_10_ deficiency is not only based on genetic failure, but also on chronic and age-related diseases such as CVDs. In this context, CoQ_10_ deficiencies have risen in CVDs, since statins, one of the most common lipid-lowering drugs used in CVD patients, diminish endogenous CoQ_10_ biosynthesis because its initial steps are shared with the cholesterol biosynthesis pathway. In this context, it has been shown that CoQ_10_ can potentially be used as a treatment to ameliorate these deficiencies. However, the existence of various CoQ_10_ formulations, together with differences in the range of doses and periods of CoQ_10_ supplementation used in the clinical studies, makes it difficult to compare them and reach a clear conclusion on the most suitable dose, effectiveness, and bioavailability of oral-administration of CoQ_10_ for therapeutic use with CVDs. A major effort is needed to reach a consensus over the use of this supplement, with the aim of including it in the clinical guidelines for treating CVD patients.

## Figures and Tables

**Table 1:** Coenzyme Q_10_ supplementation in recent studies in humans and its relation to cardiovascular disease.

Authors	Sample Size and Disease	Evidence Found	CoQ_10_ Dosage Used	Level of Evidence
Mohseni et al. (2015) [70]	52 patients with myocardial infarction	Increased serum HDL cholesterol and decreased ICAM-1 levels	200 mg/day CoQ_10_	12-week randomized, parallel group, placebo-controlled, double-blind study
Sharifi et al. (2017) [69]	63 patients with myocardial infarction	A positive effect on the physical and emotional subscales of MacNew questionnaire	150 mg/day CoQ_10_ + 1200 mg/day L-carnitine	3-month single-blind randomized
Kawashima et al. (2020) [49]	28 Patients with heart failure	An improvement in peripheral endothelial function	400 mg/day CoQ_10_ (Ubiquinol)	3-month randomized, double-blind, placebo-controlled, crossover pilot study
Sabbatinelli et al. (2020) [51]	51 patients with moderate dyslipidemia and endothelial dysfunction	An improvement in endothelium-dependent vasodilation	100 and 200 mg/day CoQ_10_ (Ubiquinol)	8-week double-blind, randomized, placebo-controlled, parallel group study
Jorat et al. (2018) [16]	526 patients with coronary artery disease	Decreased total cholesterol and increasing HDL cholesterol levels.	100 to 200 mg/day CoQ_10_	Meta-analysis (duration: 4–48 weeks)
Jorat et al. (2019) [58]	713 patients with coronary artery disease	Increased SOD and CAT, and decreased MDA	60 to 300 mg/day CoQ_10_	Meta-analysis (duration: 4–48 weeks)
Lei et al. (2017) [63]	2149 patients with heart failure	A lower mortality and improved exercise capacity	30 to 200 mg/day CoQ_10_	Meta-analysis (data not included regarding duration of supplementation)
Park et al. (2020) [77]	11 patients with peripheral artery disease	Improved brachial artery endothelial function, increased SOD, and improvement in physical functional capacity	80 mg/day MitoQ	Randomized crossover study (duration: 2 weeks)

HDL: High density lipoprotein; SOD: Extracellular superoxide dismutase; ICAM-1: Intercellular adhesion molecule 1; CAT: Catalase; MDA: Malondialdehyde.
